# Comparison of malondialdehyde levels and superoxide dismutase activity in resveratrol and resveratrol/donepezil combination treatment groups in Alzheimer’s disease induced rat model

**DOI:** 10.1007/s13205-021-02879-5

**Published:** 2021-06-13

**Authors:** Y. Lakshmisha Rao, B. Ganaraja, Aradhana Marathe, Poornima A. Manjrekar, Teresa Joy, Sheetal Ullal, Mangala M. Pai, B. V. Murlimanju

**Affiliations:** 1 Department of Anatomy, Kasturba Medical College, Manipal Academy of Higher Education, Mangalore, 575001 Manipal, Karnataka India; 2 Department of Physiology, Kasturba Medical College, Manipal Academy of Higher Education, Mangalore, 575001 Manipal, Karnataka India; 3 Department of Biochemistry, Kasturba Medical College, Manipal Academy of Higher Education, Mangalore, 575001 Manipal, Karnataka India; 4 Department of Pharmacology, Kasturba Medical College, Manipal Academy of Higher Education, Mangalore, 575001 Manipal, Karnataka India

**Keywords:** Alzheimer's disease, Lipid peroxidation, Malondialdehyde, Oxidative stress, Resveratrol, Superoxide dismutase

## Abstract

The aim of this study was to determine the malondialdehyde (MDA) level and superoxide dismutase **(**SOD) activity in colchicine induced Alzheimer’s disease **(**AD), resveratrol **(**RS) treated and RS + donepezil (DPZ) treated rat models. The objective was to compare the MDA level and SOD activity among these rat models. The present study included 3 months old male albino *Wistar* rats, which were in-house bred and weighting about 220–250 g. The rats were divided into nine subgroups which included control, sham, AD induced, RS treated and DPZ treated groups in different doses and combinations. The lipid peroxidation product for MDA in the brain homogenate was measured by estimating the levels of thiobarbituric acid reactive substance. Estimation of SOD was done by the method of autoxidation of pyrogallol by Marklund and Marklund. There was a marked increase in the MDA levels in AD induced group in comparison to the control group (*p* < 0.05). The SOD activity was higher in the RS 10 and RS 20 treated groups in contrast to the AD group (*p* < 0.05). In DPZ + RS group, there was a substantial increase in the SOD activity (*p* < 0.05). It is also observed that the RS 20 treatment group showed higher SOD activity than the RS 10 group (*p* < 0.05). This study showed that, AD induced group had elevated levels of MDA, which indicates the poor oxidative stress–defence mechanism. The RS 10 and RS 20 groups showed higher SOD activity in comparison to the AD group, which indicated the improved oxidative stress–defence mechanism. The RS + DPZ group showed higher SOD activity, indicating a synergistic effect of DPZ and RS.

## Introduction

Alzheimer's disease (AD) is an irreversible, progressive brain disorder which is often associated with the other human diseases (Surguchov 2020). The neuronal degeneration in this disorder is characterized by the cognitive dysfunction, leading into dementia (Enna and Coyle 1998). The oxidative stress was reported to be among the aetiological factors of AD (Tonnies et al. 2017). The pathophysiology involved in AD is the intra-neuronal accumulation of hyper-phosphorylated tau protein in the form of neurofibrillary tangles and extra cellular deposition of the beta amyloid plaques due to the oxidative stress (Huang et al. 2016). Brain is the most susceptible organ for oxidative stress due to its high oxygen consumption (Gomes et al. 2018). The reactive-oxygen species (ROS) are highly produced in the brain of AD patients due to the stress, which leads to the oxidative damage. The ROS accumulation along with the low antioxidant defence mechanism decreases the neuronal synaptic activity and neuro transmission, leading to the cognitive impairment (Rai et al. 2014). Thus, oxidative damage plays a crucial role in the neuronal loss and dementia. The activated microglia release several ROSs, such as superoxide radicals (O_2_), lipid peroxides (ROOH), hydroxyl radicals (OH) and hydrogen peroxide (H_2_O_2_). Excessive accumulation of these highly neurotoxic ROSs can result in lipid and protein peroxidation, and fragmentation of DNA, which later develops into the cell death (Romano et al. 2010).

Lipid peroxides are the mediators of several pathological conditions, such as neurodegeneration, inflammation and cancer. Lipid peroxidation alters the structural integrity of the cell membrane, as lipid is the major component of the cell membrane. Since lipid peroxides are highly reactive elements, they can accelerate the chain reaction of ROS formation. Through these two possible mechanisms, lipid peroxides exert their toxic effect on the cells. Eventually, lipid peroxidation regulates the non-apoptotic cell death (Gaschler and Stockwell 2017). The lipid peroxide gets degraded into two major classes like hydroxyl acids and aldehydes. Since these degradation products are also highly reactive, they are used as the important tools to quantify the lipid peroxidation. The 4-hydroxynonenal and malondialdehyde (MDA) are the degradation products of lipid peroxides, belonging to the aldehyde group, which are commonly used for the quantification of lipid peroxidation in the obtained tissue samples (Ayaala et al. 2014; Gaschler and Stockwell 2017).

Superoxide dismutase (SOD) and catalase are the enzymatic oxidants, which act as the free radical scavengers along with the other nutritional antioxidants, thereby providing the first line protection against the tissue injury, which is caused by the ROSs (Abdel Hafez et al. 2010; Pinnel 2003). SOD being present in the various intracellular compartments like cytosol, mitochondria as well as in blood plasma etc., can eliminate the super oxide radicals and the cell will be protected from the oxidative stress (Fridovich, 1995). SOD protects the cell from the oxidative damage by catalyzing the superoxide into the oxygen and hydrogen peroxide (Coimbra Costa et al. 2017). Increase in the SOD activity may reduce the lipid peroxidation (Abdel-Hafez et al. 2010) and estimation of its activity and its relation with the lipid peroxidation could help the clinicians in understanding the prognosis of the neuronal damage and to plan the treatment, accordingly. Since oxidative stress has an evidence in the pathophysiology of AD, there are several studies which have been conducted and they supported the therapeutic efficacy of the antioxidant components in the AD (Park et al. 2008; Mohan et al. 2018; Gomes et al. 2018). Extrapolating this phenomenon, the polyphenolic compounds found in the plants, which reduce the oxidative stress in these plants would provide the antioxidant support in human brain as well.

Resveratrol (RS) is a polyphenolic compound, which is majorly found in grapes and also in peanuts, pines and berries. RS is a pharmacologically proven anti-inflammatory agent, anti-cancerous agent and antioxidant, which also has anti-ageing properties (Li et al. 2019; Timmers 2012). The chemical structure of RS is P-hydroxyl group in ring A and conjugated double bond system, which is responsible for its antioxidant property. The chemical structure of RS is similar to the oestrogen diethylstilboestrol. RS enhances the anti-oxidant enzymes and is beneficial against the neuronal cell death and cell dysfunction. RS is helpful in treating diseases, such as AD, Huntington’s chorea, diabetes mellitus, cerebral ischaemia and convulsions (Rao et al. 2020). RS can reverse the colchicine induced cognitive impairment in AD model of animals (Kumar et al. 2007). RS intensifies the action of SIRT1 and related enzyme activities, which can alter the transcription profile of the neuron and improves the anti-apoptotic activities (Rao et al. 2020). DPZ is a member of second generation of acetyl-cholinesterase inhibitors and has been used in the treatment of AD. DPZ increases the hippocampal acetyl choline concentration by inhibiting the acetyl choline esterase and beta-amyloid formation. In this present research, the aim was to estimate the levels of MDA and SOD, and to compare them in the AD induced rat model and also in the RS treated subgroups. DPZ was also used as a standard treatment in the AD induced group and the effect of only RS and RS with DPZ were compared.

## Materials and methods

### Animals

3 months old male albino *Wistar* rats, which were in-house bred and weighing 220–250 g (at the start of the study) were used. Rats were fed with water and food ad libitum. Under controlled conditions of light–dark cycle, temperature (22 ± 3 °C), humidity (approximately 50 ± 10%), rats were maintained and the environment was pathogen free. The rats were housed in polypropylene cage and for bedding, the paddy husk was used. The institutional animal ethics committee’s approval was obtained before starting the experiment. This experiment was carried out in accordance with the guidelines of ‘government of India for use of laboratory animals’ (Government of India, 1999).

### Animal groups

The experiment consists of the following animal groups (*n* = 6):

Group 1: Control rats;

Group 2: Sham operated—rats with cannula implanted in the lateral ventricle;

Group 3: Colchicine induced AD model;

Group 4: AD model + RS (10 mg/kg dose), 7 days before surgery and till 7 days after surgery;

Group 5: AD model + RS (20 mg/kg dose), 7 days before surgery and till 7 days after surgery;

Group 6: AD model + RS (10 mg/kg dose), till 7 days after surgery;

Group 7: AD model + RS (20 mg/kg dose), till 7 days after surgery;

Group 8: AD model + DPZ (1 mg/kg dose), till 7 days after surgery;

Group 9: AD model + DPZ (1 mg/kg dose) + RS (10 mg /kg dose), till 7 days after surgery.

**Fig. 1:**
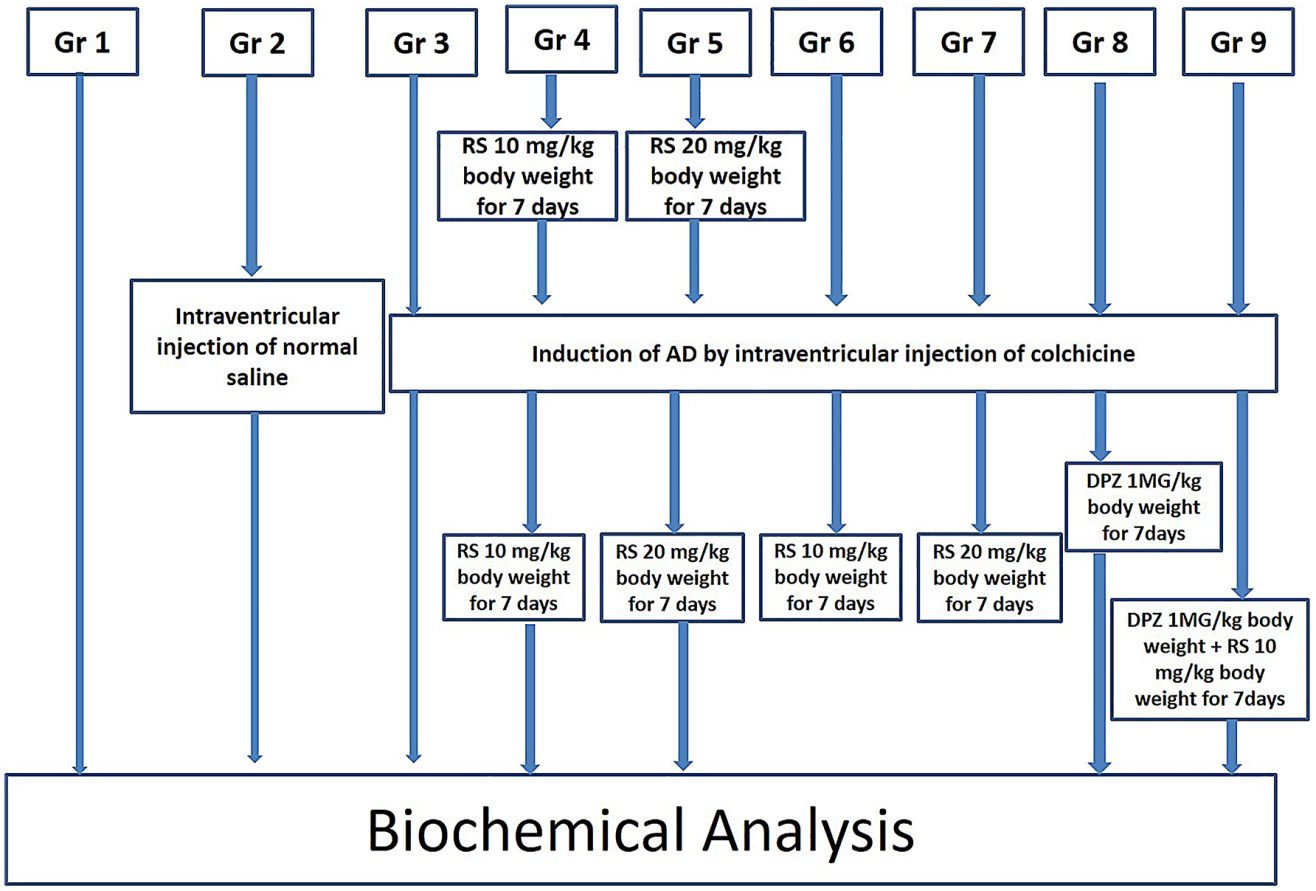
Schematic illustration of the entire experimental design of this present study

The schematic illustration of the entire experimental design of this present study is represented in Fig. 1. The normal saline was the dissolution medium used for the colchicine, RS and DPZ. The RS was administered intraperitoneally and DPZ treatment was given orally.

Colchicine was injected into the lateral ventricle by the stereotaxic surgery in the groups 3–9. RS treatment was started one week before the surgery, in the groups 4 and 5, which was continued till 7 days after the surgery. For groups 6 and 7, RS treatment was started from the next day of surgery. In group 8, DPZ treatment was started from the next day of surgery and continued for 7 days. In group 9, DPZ + RS was given for seven days starting from the next day of surgery. In all these groups, brain was removed by decapitating the animals on the next day after the completion of treatment.

### Chemicals

RS was purchased from Tokyo chemical industry Co. Ltd., Tokyo, Japan (product number, R0071, very pale yellow powder, 99.9% purity). Colchicine (catalogue number 9754) was obtained from Sigma Aldrich, Bangalore and remaining chemicals, reagents were from HPLC or analytical grade (Sigma, St. Louis, Mo.).

The dosage of RS was taken with reference of previous studies (Wiciński et al. 2020; Sharma and Gupta 2002). According to the literature, the dosage of DPZ ranges from 0.375 to 0.75 mg/kg/day (Hernandez et al. 2006) to 3–10 mg/kg/day (Shin et al. 2018) in different studies carried out in rat model. In this present study, the dose was standardized after the pilot study in our laboratory and 1 mg/kg has shown positive results.

### Surgery and intracerebro-ventricular administration of colchicine

The surgical procedure involved implantation of a cannula into the lateral ventricle, as per the previous study by Madhyastha et al. (2002). The rats were subjected to general anaesthesia with sodium pentobarbital (40 mg/kg, i.p). The lateral ventricle was approached on the right side by using the standard stereotaxic coordinates (antero-posterior position—0.8 mm posteriorly from the bregma, ± 2 mm laterally from the bregma and 3.2 mm deep to the skull surface). The skull cap was drilled carefully up to the level of dura mater, without damaging the nervous tissue. A sterile needle, which is connected to the cannula carrier was slowly introduced into the lateral ventricle at the depth of 3.2 mm. Later, the needle was connected to Hamilton micro syringe for the administration of colchicine. Colchicine, 15 µg in a volume of 5 µl was injected through the Hamilton micro syringe which was placed in Harvard apparatus infusion pump. Thereafter the needle was gently removed and the wound was closed by using the dental cement. Special care was taken to prevent the infection during the post-surgery period.

The induction of AD was confirmed by the behavioural studies and neuronal assay. The comparison was performed between the AD group and control group. The behavioural studies involved open-field test and the active avoidance test. The open field test showed reduced exploratory activities like grooming and rearing. Active avoidance test showed decreased retest score and retention score. The results of these tests gave inference about the impairment of cognition and memory, which suggested the induction of the AD model. The neuronal assay was showing decrease in the neurons in the frontal cortex and hippocampal regions.

### Biochemical analysis

In all the groups of this present study, MDA levels were measured to estimate the level of lipid peroxidation. The SOD activity was also measured to see the antioxidant activity in the brain. Later both the MDA and SOD were correlated to study the relation between the lipid peroxidation and antioxidant activity.

### Tissue processing

Twenty-four hours after the end of treatment, biochemical analysis was performed. The animals were sacrificed by the cervical decapitation. Brain was removed rapidly and rinsed and homogenized with 0.1 M/L phosphate buffer saline (pH 7.4). Each homogenate sample was centrifuged at 10,000×*g* for 20 min at 4 °C, and aliquots of supernatant were separated and used for the following biochemical estimations.

### Lipid peroxidation

The lipid peroxidation product in brain homogenate was measured by the estimation of malondialdehyde (MDA), through the estimation of thiobarbituric acid reactive substances (TBARS) by the method described by Buege and Aust (Fleischer and Packer 1978). One millilitre of supernatant was precipitated with 2.5 ml of ice-cold trichloro acetic acid (TCA). The samples were centrifuged at 3000*g* for 10 min. To 2 ml of this supernatant, 0.67% of thiobarbituric acid (TBA) was added and kept in boiling water bath for 45 min, which was immediately chilled. The pink chromogen developed was read immediately at 532 nm by using a systronic-117 UV–visible spectrophotometer. TBARS concentration was calculated by using the molar extinction coefficient of chromophore (1.56 × 10^5^(mol/l)^−1^ cm^−1^) and the values are expressed in µmoles/gm. protein.

### Estimation of superoxide dismutase (SOD)

Estimation of superoxide dismutase (SOD) was performed by the method of autoxidation of pyrogallol by Marklund & Marklund. For the test sample, 0.1 ml of tissue homogenate is treated with 2.5 ml of Tris–HCl buffer of pH 8.2, 0.1 ml of 1 mM EDTA (ethylene diamine tetra acetic acid), 0.5 ml of 1 mM DTPA (diethylene triamine penta acetic acid) and 0.1 ml of 0.02 mM pyrogallol. Kinetic end point method was used, where change in the autoxidation of pyrogallol was observed for 3 min for each sample at 420 nm. The control sample was run in the same way, without taking tissue homogenate but taking the 2.6 ml of Tris–HCL buffer.

### Protein estimation

The total protein concentration for tissue was calculated by Lowry’s method (using Folin–Ciocalteu reagent). Inhibition of auto-oxidation of pyrogallol happened at an alkaline pH by the superoxide dismutase enzyme which was present in the sample. One unit of the enzyme activity is defined as the amount which produced 50% of inhibition of pyrogallol autoxidation under the standard assay conditions. The unit is expressed as U/mg of tissue protein.

### Statistical analysis

The ‘EZR’ software was used to perform the statistical analysis. One-way ANOVA and post hoc tests were applied to compare the SOD and MDA levels in the different subgroups of this study. The ‘*p*’ value was adjusted by the Bonferroni method. To correlate the SOD and MDA levels, Pearson’s correlation test was conducted. The comparisons were considered as statistically significant if the ‘*p*’ value is smaller than 0.05.

## Results

**Fig. 2:**
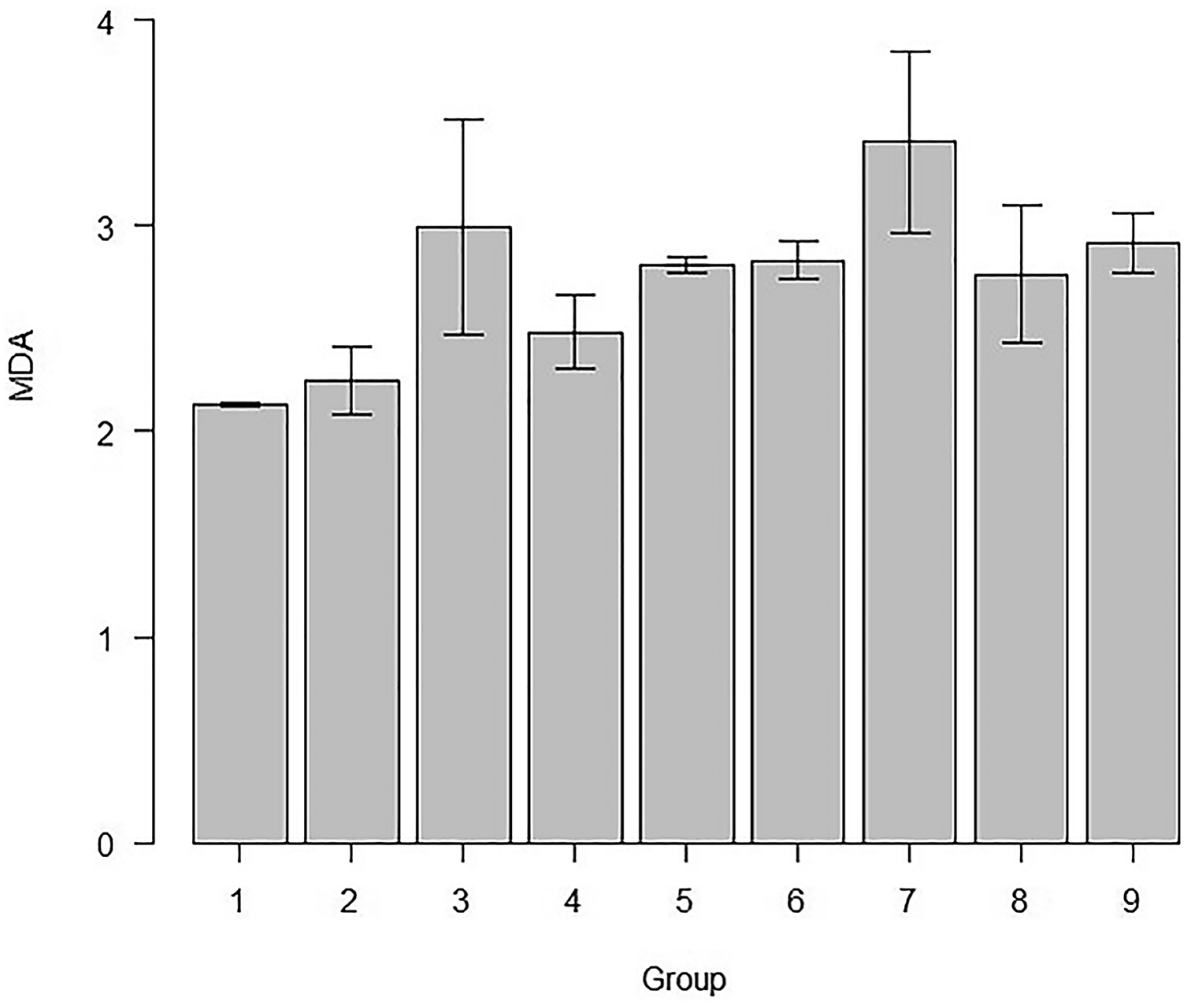
Comparison of MDA levels of all the study groups of this study (mean ± SD)

**Fig. 3:**
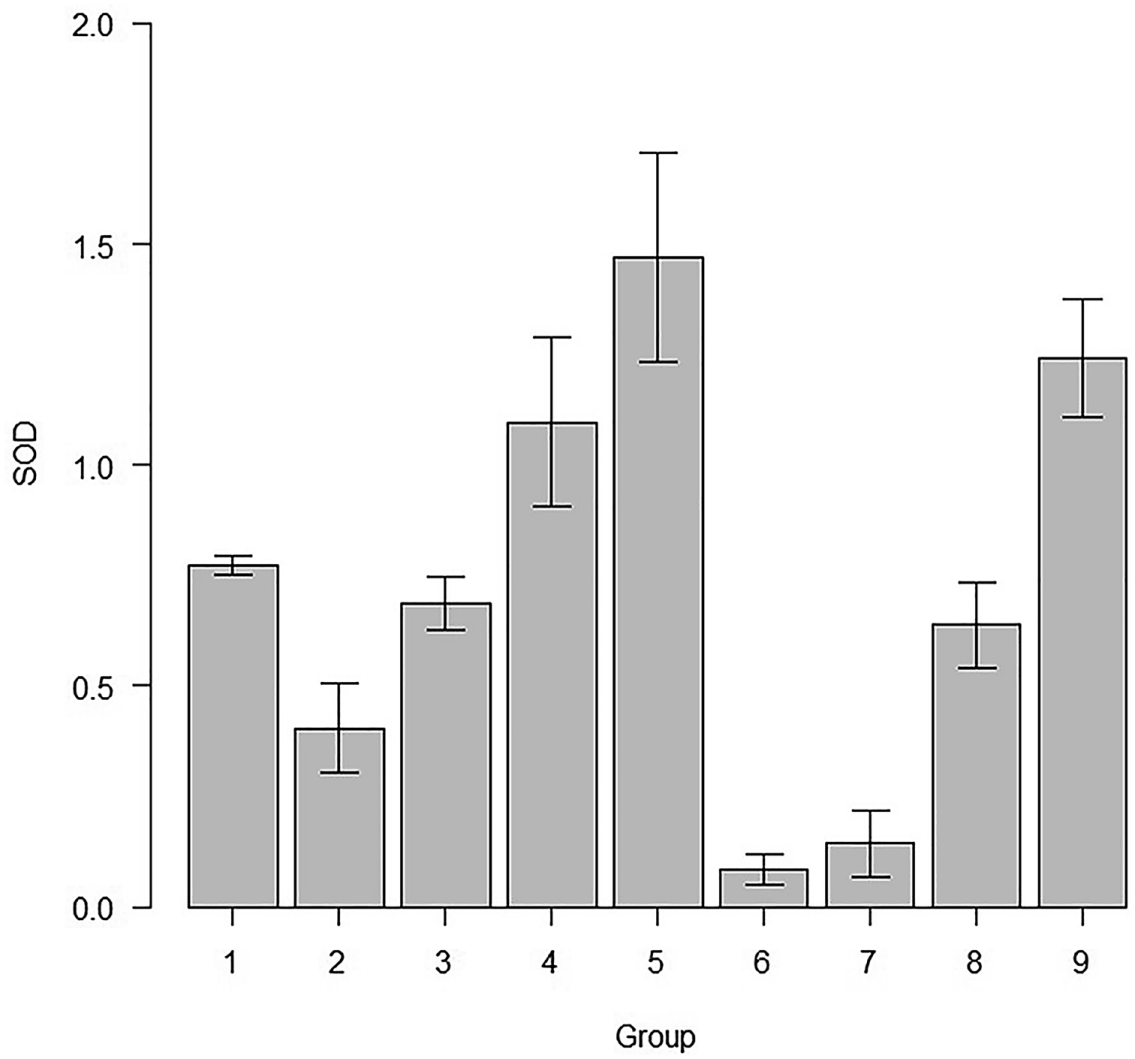
SOD activity in the various subgroups in this study (mean ± SD)

MDA levels and the SOD activity, among various groups of this study is represented in Table 1. The comparison of MDA levels and SOD activity among different subgroups were compared each other and the ‘p’ values are given in Tables 2 and 3. The mean and standard deviation of MDA levels of all the study groups are represented in Fig. 2. The mean SOD activity and the standard deviations of the subgroups of the study are represented in Fig. 3.

**Table 1:** Levels of MDA and SOD levels in the various study groups

Groups	MDA	SOD
Control (1)	2.13 ± 0.01	0.77 ± 0.02
Sham (2)	2.24 ± 0.16	0.40 ± 0.10
AD (3)	2.99 ± 0.52	0.68 ± 0.06
RS 10 (4)	2.48 ± 0.18	1.09 ± 0.19
RS 20 (5)	2.81 ± 0.03	1.47 ± 0.23
RS 10/10 (6)	2.83 ± 0.09	0.08 ± 0.03
RS 20/20 (7)	3.4 ± 0.44	0.14 ± 0.07
DPZ (8)	2.76 ± 0.33	0.63 ± 0.09
RS + DPZ (9)	2.91 ± 0.14	1.24 ± 0.13

**Table 2:** ‘*p*’ values of pairwise comparison of the subgroups for the MDA level

Group	1	2	3	4	5	6	7	8
2	1							
3	0.000075*	0.00082*						
4	1	1	0.08145					
5	0.00354*	0.03236*	1	1				
6	0.00224*	0.0211*	1	1	1			
7	0.000000011	0.00000012	0.45958	0.00002	0.01745	0.02685		
8	0.0085	0.07265	1	1	1	1	0.00741	
9	0.00039	0.004	1	0.308	1	1	0.12	1

One-way-ANOVA and post hoc test; statistical significance, *p < 0.05; *p* value adjustment: Bonferroni method

**Table 3:** ‘*p*’ values of pairwise comparison of the subgroups for the SOD activity

Group	1	2	3	4	5	6	7	8
2	0.00025*							
3	1	0.01138*						
4	0.00183*	0.000000000073*	0.000035*					
5	0.000000000051*	0.0000000000000002*	0.000000000003*	0.00017*				
6	0.000000000088*	0.00221*	0.0000000042*	0.0000000000000002*	0.0000000000000002*			
7	0.0000000012*	0.02643*	0.000000061*	0.0000000000000016*	0.0000000000000002*	1		
8	1	0.0806	1	0.0000037*	0.00000000000018*	0.000000039*	0.00000058*	
9	0.0000022*	0.00000000000016*	0.000000038*	1	0.09091	0.0000000000000002*	0.0000000000000002*	0.0000000042*

One-way ANOVA and post hoc test; statistical significance, *p < 0.05; *p* value adjustment: Bonferroni method

### MDA activity

There was obvious elevation in the MDA levels in the AD group in comparison to the control group (*p* < 0.05). The MDA level is increased in AD group in comparison to the Sham group (*p* < 0.05). The MDA level was high in the RS 20/20 group in comparison to the RS 10, RS 20 and RS 10/10 groups (*p* < 0.05). The statistical significance was not observed in the comparison of the other groups each other (p > 0.05).

### SOD activity

In this present study, RS 10, RS 20, RS 10/10 and RS 20/20 treated groups showed significantly higher SOD activity, in comparison to the control group (*p* < 0.05). In contrast, SOD activity was increased only in RS 10, RS 20 and RS + DPZ groups when compared with the sham group (*p* < 0.05). There was statistically significant increase in the SOD activity of RS 10, RS 20 and RS + DPZ groups, in comparison to the AD group (*p* < 0.05). However, the SOD activity was decreased in the RS 10/10 and RS 20/20 groups in comparison to the AD group (*p* < 0.05). The SOD activity was increased in the RS 20 group than the RS 10 group (*p* < 0.05). However, the SOD activity was decreased in the RS 10/10 and RS 20/20 groups in comparison to the RS 10 group (*p* < 0.05). Even RS 20 group showed significant increase in the SOD activity in comparison to the RS 10/10 and RS 20/20 groups (*p* < 0.05). In DPZ + RS set, SOD activity was elevated than the RS 10/10 and RS 20/20 groups (*p* < 0.05). However, there was no statistically significant difference observed between the RS + DPZ group and RS 10, RS 20 groups with respect to the SOD activity (*p* > 0.05).

### Comparison of the SOD activity and MDA level

**Fig. 4:**
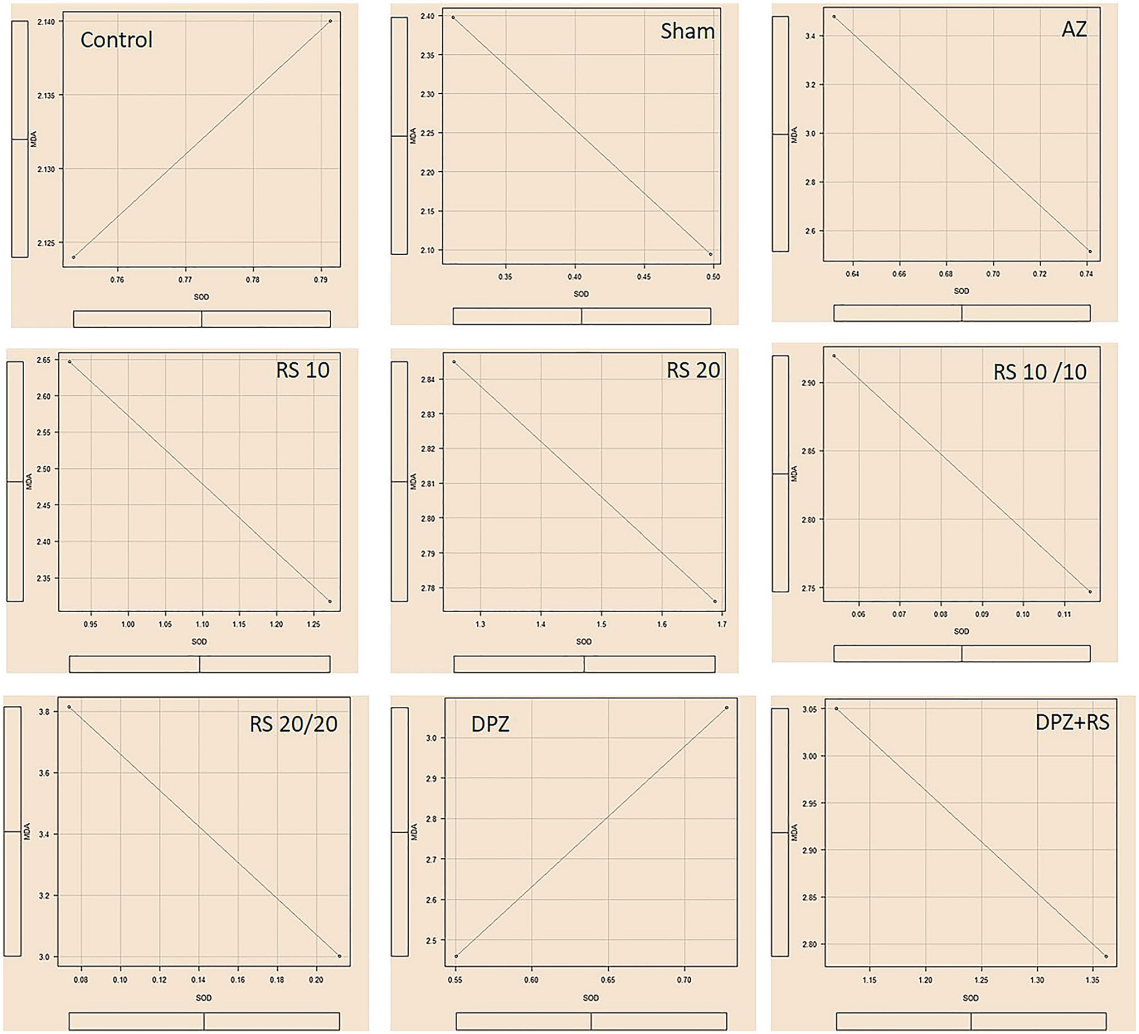
Pearson’s correlation graphs of all the subgroups of this present study

The Pearson’s correlation test was done to correlate the SOD activities with MDA levels in the groups. It was observed that, in all the groups, there was a highly significant negative correlation (‘*r*’ = − 1) except for the DPZ group (‘*r*’ =  + 1) (Fig. 4). The correlation observed in all the groups is represented in Fig. 4.

## Discussion

The present research observed that RS can protect the brain tissue from the oxidative damage through its antioxidant property and also by activating the existing antioxidants in the brain. The preferable dose of RS to reverse the neuronal damage was 20 mg per kg body weight. Even though 10 mg/kg body weight dose of RS was effective, not as effective than the 20 mg/kg body weight dose. The mechanism of action and dosing effects was beyond the purview of this study. It may be hypothesized that the therapeutic window of RS may be around 20 mg/kg. When RS was given as prophylactic dose, there was no significant protection from the lipid peroxidation. Hence, the prophylactic therapy of RS may not be helpful in the oxidative stress conditions. Our study has proved that, when RS was administered with the combination of DPZ, the antioxidant activity of the brain will be more effectual, which can prevent the neuro apoptosis. RS is a pharmacologically proven antioxidant and may also act by increasing the SOD activity. However, as far as the authors knowledge goes, DPZ has not yet been reported to have antioxidant property. The heightened antioxidant property of RS when combined with DPZ could be due to the synergistic neuroprotective effect of the two compounds, which may result in higher SOD activity. For the synergistic action, the two drugs that are combined should have different mechanisms of action. When the two drugs have similar effects, but by differing mechanisms, the effect is always potentiated. The same thing seems to have happened with RS and DPZ. Hence, the rationale of the combination in this present study. Moreover, in the RS and DPZ combination group, there is more SOD level than the control group. The present study also observed the lesser level of SOD activity in the sham group as compared to the AD group. This is because, as the AD group was treated with colchicine, this resulted in the oxidative stress due to which SOD activity was reduced. The novelty of the present study is that, the combination of RS and DPZ was used. This is the first report in the literature, which used RS and DPZ combination, in a colchicine induced AD rat model. Since AD is a multifactorial disorder, multi-targeted drugs would prove to offer better therapeutic strategy in the treatment of AD patients.

In AD, extracellular accumulation of Aβ will generate the hydrogen peroxide, lipid peroxides and degradation products such as MDA (Xia et al. 2017). It was reported that, over expression of SOD has reduced the lipid peroxidation in an ischaemic brain damage rat model (Keller et al. 1998). The association amongst the oxidative trauma and lipid peroxidation is widely studied in the transient brain injury. The lipid peroxidation is detected by the estimation of its end products like MDA, 4- HNE and isoprostanes in the brain tissue samples (Anthonymuttu et al. 2016). Since lipid peroxidation and oxidation stress is one of the pathophysiology of AD, antioxidants may be used in the treatment of AD. However, Lloret et al. (2019) opined that vitamin E could not stop the progression of AD. Since lipid peroxidation starts in the early-stages of AD, Pena-Bautista et al. (2019) opined that supplementation with antioxidants in the early-stage of AD could prevent the advancement of the disease. RS, by activating the SIRT1 can regulate the inflammation and oxidative injury (Wang et al. 2017). RS is a direct scavenger of the free radical production. The chemical structure of the RS has contributed its antioxidant property as it contains three hydroxyl groups in it. Since antioxidant property of a compound increases along with the increase in –OH group in it, RS could be an effective antioxidant. The hydroxyl groups of the RS will help to chelate the metals, thereby preventing the ROS generation (Sedlak et al. 2018). In animal model of Aβ encouraged neurodegeneration, RS has shown its efficacy by lowering the activity of the oxidative stress inducing enzymes along with the induction of antioxidant enzymes like SOD, catalase etc. RS has reduced the Aβ induced lipid peroxidation (Rege et al. 2014). Kumar et al. (2007) has reported that, in colchicine induced cognitive impairment in rat models, RS was able to reverse the condition to a greater extent. They also reported the decrease in the level of MDA, when resveratrol was given for 25 days orally. Li et al. (2014) observed that RS stopped the p-53 induced apoptosis by expressing SIRT1, which induces the manganese SOD. RS reduced the lipid peroxidation, interleukins and systolic blood pressure, in rats fed with high quantity of fat (da Fonseca et al. 2020). In vitro oxidative stress to the human erythrocytes, which was given by incubating with tertbutylhydroperoxide (t-BHP) increased the MDA level, which showed the lipid peroxidation. But the presence of trans RS in the incubating media had decreased the MDA level (Pandey and Rizvi, 2009). Vitaglion et al. (2009) observed the hepato-protective action of trans RS, against the liver lipid peroxidation, which was induced by the carbon tetrachloride (CCl_4_). They used the liver MDA as lipid peroxidation marker. Dietary supplement of RS has reduced the MDA level in various tissues like liver, heart, brain and testes in ethanol induced lipid peroxidation. This shows the protective property of RS alongside the oxidative stress in various organs (Kasdallah-Grissa et al. 2006). Ro et al. (2020) opined that RS will protect the brain from lipid peroxidation in cerebral ischaemic rats. They have observed the markedly lowered MDA level and increased antioxidants like SOD and catalase in the brain, when treated with RS 20 mg/kg body weight. RS prevented the oxidative stress in the muscle, which was induced by the exercise, by preventing the lipid peroxidation (Nasiri et al. 2020). As per Kong et al. (2019), high dose of RS (40 mg/kg for 15 days) has reduced the MDA level and increased the SOD activity. The lower dose of RS (20 mg/kg for 15 days) has reduced the MDA level better than the higher dose group (Kong et al. 2019). In rat model of diabetes associated dementia, DPZ and metformin combination has modulated the antioxidant status of the brain by decreasing the MDA level and increasing the SOD activity (Obafimi et al. 2020).

## Conclusion

Colchicine induced the oxidative stress, which was leading to the increased lipid peroxidation. This was proved by the elevated MDA levels in this AD rat model. However, RS decreased the lipid peroxidation and increased the SOD activity, which indicated the stress–defence mechanism. The neuroprotective effect of RS is produced by the reduced oxidative mutilation in the brain tissue in the treated group. RS + DPZ group showed higher SOD activity, indicating a synergistic effect of DPZ and RS.
